# Scavenger receptor class B type I genetic variants associated with disease severity in chronic hepatitis C virus infection

**DOI:** 10.1002/jmv.28331

**Published:** 2022-11-30

**Authors:** Victoria L. Arandhara, Charles Patrick McClure, Alexander W. Tarr, Sally Chappell, Kevin Morgan, Thomas F. Baumert, William L. Irving, Jonathan K. Ball

**Affiliations:** ^1^ School of Life Sciences, The University of Nottingham Queen's Medical Centre Nottingham UK; ^2^ Wolfson Centre for Global Virus Research, The University of Nottingham Queen's Medical Centre Nottingha UK; ^3^ NIHR Nottingham Biomedical Research Centre at the Nottingham University Hospitals NHS Trust and University of Nottingham Nottingham UK; ^4^ Institut de Recherche sur les Maladies Virales et Hépatiques Université de Strasbourg, Inserm Strasbourg France; ^5^ IHU Strasbourg, Pôle hépato‐digestif, Hôpitaux Universitaires de Strasbourg Strasbourg France

**Keywords:** HCV, liver disease, rs5888, scavenger receptor class B type 1, single nucleotide polymorphisms, SR‐BI

## Abstract

Analysis of host genetic polymorphisms is an increasingly important tool for understanding and predicting pathogenesis and treatment response of viral diseases. The gene locus of scavenger receptor class B type I (SR‐BI), encoding a cell entry factor and receptor for hepatitis C virus (HCV), contains several genetic polymorphisms. We applied a probe extension assay to determine the frequency of six single nucleotide polymorphisms (SNPs) within the SR‐BI gene locus in 374 individuals with history of HCV infection. In addition, SR‐BI messenger RNA (mRNA) levels were analyzed in liver biopsy specimens of chronically infected HCV subjects. The rs5888 variant allele T was present at a higher frequency in subjects with advanced fibrosis (*χ* ^2^, *p* = 0.016) and after adjusting for age, duration of infection and alcohol intake as confounding factors. Haplotype analysis of SNP frequencies showed that a haplotype consisting of rs61932577 variant allele C and rs5888 variant allele T was associated with an increased risk of advanced liver fibrosis (defined by an Ishak score 4−6) (adjusted odds ratio 2.81; 95% confidence interval 1.06−7.46. *p* = 0.038). Carriers of the rs5888 variant allele T displayed reduced SR‐BI mRNA expression in liver biopsy specimens. In conclusion the rs5888 polymorphism variant is associated with decreased SR‐BI expression and an increased risk of development of advanced fibrosis in chronic HCV infection. These findings provide further evidence for a role of SR‐BI in HCV pathogenesis and provides a genetic marker for prediction of those infected individuals at greater risk of developing severe disease.

## 1 INTRODUCTION

The consequences of infection with hepatitis C virus (HCV) differ considerably between individuals and the spectrum of liver disease in chronic HCV infection can vary widely. Host factors such as age, gender, duration of infection, alcohol consumption, and route of transmission may influence disease progression.^1^, ^2^, ^3^, ^4^, ^5^ There is also evidence that host genetic factors influence the course of infection and development of liver disease,^6^, ^7^ the most noticeable being the influence of genetic variation within the interferon lambda locus on infection and treatment outcome (reviewed in).^8^, ^9^ Additional polymorphisms within genes encoding cell surface proteins, including low density lipoprotein receptor (LDL‐R)^10^ and apolipoprotein E^11^ are associated with severity of fibrosis, viral clearance, overall inflammation or treatment response.^10^ The scavenger receptor class B type I (SR‐BI), which is primarily expressed in the liver and in steroidogenic tissues,^12^, ^13^, ^14^, ^15^ serves as one of several host surface molecules involved in HCV cell entry.^16^ Its exact role in entry has not been fully elucidated, but direct binding to the E2 envelope protein is thought to facilitate subsequent binding to the major receptor, CD81,^17^, ^18^, ^19^ and SR‐BI‐specific monoclonal antibodies inhibit HCV infection in cell‐based and animal models.^20^, ^21^

SR‐BI plays an important role in regulating plasma HDL levels and is a major determinant of HDL cholesterol levels.^22^, ^23^, ^24^, ^25^, ^26^ In selective lipid uptake, HDL binds to the cells but only the lipid part of the particle enters the cells via an unknown mechanism that does not require endocytosis of the lipoprotein particle.^27^ The SR‐BI gene locus contains several genetic polymorphisms^23^ and there is evidence that these are associated with plasma lipid profiles and the development of atherosclerosis and coronary artery disease.^6^, ^23^, ^28^, ^29^, ^30^, ^31^, ^32^, ^33^, ^34^, ^35^, ^36^, ^37^, ^38^, ^39^, ^40^ Previous studies have shown that some common SR‐BI polymorphisms are associated with differential HCV treatment outcome and viral load.^41^, ^42^ In addition, naturally occurring, but very rare SR‐BI coding mutations (specifically S112F and T175A) have reduced HCV entry function.^42^, ^43^, ^44^ Despite the association between SR‐BI polymorphisms and key facets of HCV replication and biology, their potential impact on disease severity has not been defined. To address this shortfall, we used archived DNA extracts obtained from the extensively characterized Trent HCV cohort^45^ to investigate the prevalence of relatively high frequency polymorphisms in individuals with severe versus mild HCV‐induced liver disease. We also defined the relationship between SR‐BI polymorphisms and SR‐BI messenger RNA (mRNA) expression in liver biopsies from chronically infected HCV subjects using real‐time  polymerase chain reaction (PCR).

## 2 PATIENTS AND METHODS

### 2.1 Human subjects

Archived samples from patients with a history of HCV infection were obtained from the Trent HCV study cohort, which was established in 1991 with the aim of studying the epidemiology and natural history of HCV infection.^1^, ^4^, ^46^ All patients referred to one of the participating clinics were invited to enroll in the cohort study with informed consent following approval by the local ethics committee. HCV antibody positive subjects (third‐generation enzyme‐linked immunosorbent assays, Ortho; Sanofi) were divided, based on historic diagnostic testing protocols, into those who were negative (acute resolved infection) or positive (chronic infection) for HCV RNA (Amplicor reverse‐transcription PCR assay; Roche). Diagnostic liver biopsy specimens were scored using the Ishak modified histological activity index^47^ and grouped into severe liver disease, defined by the presence of advanced fibrosis (Ishak fibrosis stage 4−6), and mild liver disease (Ishak fibrosis stage 0−3). Following the Trent Hepatitis C Cohort Study assessment and management protocol^1^ patients are offered repeat liver biopsies at 2‐year intervals. Subsequently several patients in this study had undergone more than one liver biopsy. For this study, the highest fibrosis score achieved was recorded irrespective of whether this was on a first or subsequent biopsy. Risk factors for acquisition of the virus were categorized as injecting drug use, blood product transfusion, other known risk factor (e.g., tattoo and body piercing) and no known risk factor. The duration of infection at the time of biopsy was estimated assuming that the first exposure to risk was the date of infection. At each clinical visit the average weekly alcohol intake since the previous visit was recorded. Clinical, ApoE and interferon lambda genotype data for some of these patients have previously been described.^4^, ^11^, ^48^

### 2.2 Genotyping of SR‐BI polymorphisms

To assess the possible relationship between SR‐BI polymorphisms and the outcome of infection with HCV, four single nucleotide polymorphisms (SNPs) present within the gene locus were selected, based on previous evidence of an association with either lipid metabolism and/or HCV outcomes.^23^, ^33^, ^36^, ^39^, ^41^, ^42^, ^49^ In addition, two control SNPs, where no associations with disease had been reported, were included in the analysis (Table 1). DNA was extracted from 200 µl of either whole blood or plasma using Qiagen DNA blood mini kit according to the manufacturer's protocol. Oligonucleotide primer pairs (Table 2) were derived from a previous study^23^ or mapped to human contiguous sequences (Genbank Accession AC126309 and AC073593) flanking each SNP of interest. Primers were designed to amplify a 200−300 nucleotide region surrounding the SNP of interest. Aliquots of DNA (1 μl) were used as templates in PCR reactions containing 0.2 µM of each primer pair, 200 μM dinucleotide triphosphates (dNTPs) and 0.625 U Hot Star Taq (Qiagen,) in 1 x PCR buffer in a 25 μl reaction volume. The amplification cycle parameters were an initial denaturation step of 94°C for 10 min followed by 35 cycles of denaturation (94°C, 50 s), annealing (55°C, 30 s) and extension (72°C, 1 min) and an extended elongation step (72°C, 5 min). PCR products were purified by treatment with shrimp alkaline phosphatase (SAP and GE Healthcare) and exonuclease I (Exo I, New England Biolabs) to remove unincorporated dNTPs and primers.

**Table 1:** SR‐BI SNPs under study

SNP	Alleles	Highest minor allele frequency^a^	cDNA position	Amino acid	Phenotype	Reference
rs4238001	G > A	6%	4	G2S	Increased MI risk; Decreased HDL/Elevated LDL (in males); Decreased SR‐BI protein expression	23, 36, 39, 49
rs5889	C > T	11%	502	G167G	Elevated HDL; Decreased coronary artery disease;	70
rs5892	C > T	4%	905	F301F	‐	
rs5888	C > T	32%	1050	A350A	Decreased LDL/Increased BMI (in women);	23, 37, 40
rs61932577	C > T	14%	‐	Intron	‐	
rs2293440	T > C	24%	‐	Intron	Altered HDL levels	71

^a^ Minor allele frequency as recorded in the 1000 Genomes Project Phase 3, Ensembl database.^46^

**Table 2:** Characteristics of patients grouped by HCV status and Ishak fibrosis stage

	HCV RNA		HCV Ishak Fibrosis	
	Negative (resolved)	Positive (chronic)	Mild (0−3)	Severe (4−6)
	*n* = 107	*n* = 267	*n* = 160	*n* = 92
Gender				
Male	67 (63%)	183 (69%)^a^	111 (69%)	63 (69%)^a^
Female	38 (35%)	81 (30%)	49 (31%)	26 (28%)
Unknown	2 (2%)	3 (1%)	0 (0%)	3 (3%)
Risk factor				
Injecting drug user	40 (37%)	151 (56%)	106 (66%)	36 (39%)
Blood product transfusion	4 (4%)	45 (17%)	27 (17%)	15 (16%)
Other known risk factor	4 (4%)	15 (6%)	6 (4%)	9 (10%)
No known risk factor	1 (1%)	33 (12%)	14 (9%)	17 (19%)
No risk factor data	58 (54%)^b^	23 (9%)	7 (4%)	15 (16%)
Mean (SD) age at Biopsy (year)			39 (10)	50 (11)^c^
Mean (SD) duration of Infection (year)			17 (8)	23 (10)^c^
Median weekly alcohol intake (range)			4 (0−65)	0 (0−60)^d^

^a^ NS (*χ* ^2^ test);

^b^ Risk factor data was not routinely collected from HCV RNA negative patients;

^c^ *p* < 0.001 (unpaired *t*‐test);

^d^ *p* = 0.001 (Mann−Whitney *U* test).

Allelic composition was determined using the commercially available ABI PRISM Mulitplex SNaPShot Reaction kit (ThermoFisher). SNaPshot primers (Supporting Information: Table 1) were designed adjacent to the SNP of interest and 5′ polyA tails added to produce primers of differing lengths to allow discrimination of individual SNPs in a multiplex reaction. The SNaPshot reaction was prepared according to the manufacturer's protocol. Samples were resolved using an Applied Biosystems Genetic analyzer according to the manufacturer's protocol.

### 2.3 Sequencing of PCR products

Each allelic variant was confirmed by direct sequencing of PCR products that were used in the SNP analysis using the ABI Prism Dye Terminator Cycle Sequencing Ready Reaction Kit (Applied Biosystems) according to the manufacturer's protocol. Samples were selected for sequencing at the first detection instance of each SNP genotype.

### 2.4 RNA extraction from liver biopsies

Percutaneous needle liver biopsies were obtained from subjects attending the liver clinic at Lincoln City Hospital between 1999 and 2003, consented to join the HCV Trent Study cohort.^1^, ^4^ Biopsies were snap frozen in liquid nitrogen and nucleic acids using TRIzol reagent (Invitrogen) according to the manufacturer's guidelines. 1 ml TRIzol was added to the frozen liver biopsies and the tissue homogenized using a Ribolyser (Bio‐Rad) at speed 6 for 40 s, repeating until the biopsy was homogenized, before nucleic acid extraction. Reverse transcription was performed using ThermoScript RT‐PCR System (ThermoFisher) according to the manufacturer's guidelines using random hexamers for priming.

### 2.5 Quantitative real‐time PCR

Investigation of mRNA copy numbers in liver biopsy specimens was performed using Brilliant SYBR Green Quantitative PCR Master Mix (ThermoFisher) according to the manufacturer's instructions. HPRT was amplified as a reference gene using the following primers: HPRTA, 5′ GACCAGTCAACAGGGGACAT 3′ and HPRTB 5′ CGACCTTGACCATCTTTGGA 3′. SR‐BI mRNA was amplified using primers SR‐BIF 5′ GAAACTGCAGCTGAGCCTCT 3′ and SR‐BIR 5′ ATTTCTCTTGGCTCCGGATT 3′. A standard curve of cycle threshold (*C*_t_) plotted against a standard of known copy number was used to interpolate the copy number of unknown samples using the C_t_ for each sample. Relative copy numbers of the reference gene HPRT and SR‐BI were calculated for each unknown sample for comparison.

### 2.6 Statistical analyses

SPSS version 12.0.1 was used for statistical analyses. Variables were compared using the unpaired *t*‐test for two independent groups for normally distributed variables and the Mann−Whitney *U* test for non‐normally distributed variables. SR‐BI genotype allele frequencies were estimated by allele counting and deviations from Hardy‐Weinberg equilibrium were assessed for the whole cohort. The statistical significance of associations between genotype and total allele frequency distribution for each SNP between patient groups was assessed using *χ* ^2^‐tests. Logistic regression analysis was performed to adjust for confounding factors. A *p* value of <0.05 was considered statistically significant. Haplotype analysis and linkage disequilibrium (LD) analysis was performed using the COCAPHASE software utility of the UNPHASED suite of programs for association analysis for multilocus haplotypes (https://gaow.github.io/genetic-analysis-software/u/unphased/)^50^ and using the Ensembl Genome Browser.^46^ To compare the SR‐BI mRNA levels in liver biopsy samples the number of copies of SR‐BI per HPRT mRNA copy was calculated by dividing the number of copies of SR‐BI by the number of copies of the reference gene HPRT. The copies of SR‐BI per HPRT were compared between genotypes for rs4238001, rs61932577 and rs5888 and the significance of differences between the median values determined using Kruskal‐Wallis test for three or more groups and the Mann–Whitney *U* tests for two groups. Analyses were performed using GraphPad Prism version 9 for Windows (GraphPad Software). To assess inter‐ and intra‐assay variation the coefficient of variation (CV) of *C*_t_ values was calculated as (standard deviation/mean) x 100.^51^ The CV for HPRT *C*_t_ values was less than 3.21% for standards (range 0.81% to 3.21%) and less than 7.7% for unknown samples (range 0%−7.7%). For SR‐BI the CV of the *C*_t_ values was less than 11.24% for standards (range 0.95%−11.24%) and less than 8.94% for unknown samples (range 0.37%–8.94%). CVs for intra‐assay variation from two intra‐assay repeats were below 7% for both HPRT and SR‐BI unknown samples and less than 2% for standard *C*_t_ values.

## 3 RESULTS

### 3.1 Patient characteristics

A total of 374 subjects with a history of HCV infection were studied. Of these, 107 (28.6%) had acute resolved infection and 267 (71.4%) had chronic infection. Histological assessment of liver biopsies was available for 252 chronically infected subjects of which 92 (36.5%) had severe liver disease (Ishak fibrosis stage 4−6) and 160 (63.5%) had mild liver disease (Ishak fibrosis stage 0−3). Characteristics of patients grouped by HCV status and Ishak fibrosis stage are given in Table 2. Several host factors may influence the degree of liver fibrosis in chronic infection with HCV including gender, alcohol intake and duration of infection.^3^ No associations were found between gender and HCV status (*χ* ^2^ test, *p* = 0.33) or severity of liver disease (*χ* ^2^ test, *p* = 0.89). The duration of infection was estimated for 187 subjects from whom risk factors were known and was estimated from the first exposure to that risk factor. The mean estimated duration of infection and mean age at the time of biopsy procedure were higher in the subjects with severe liver disease (unpaired *t*‐tests, *p* < 0.001). The median average weekly alcohol intake (units) since the previous clinical visit was higher in the group of subjects with mild liver disease compared to those with severe liver disease (*n* = 252, Mann−Whitney *U*, *p* = 0.001). The cohort was infected with genotypes 1−5, with genotypes 1 and 3 the most prevalent in both groups (Data not shown). Genotype prevalence did not differ significantly between the groups (*χ* ^2^ test, *p* = 0.122).

### 3.2 Genotyping and distribution of SR‐BI polymorphisms

To determine possible associations with SR‐BI polymorphisms and the outcome of HCV infection, the allele frequencies at six polymorphic sites were assessed. Four of the SNPs were in exon sequences and two in introns (Table 1). Allele counting revealed that the frequency of minor allelic variants defined by rs5889, rs2293440, and rs5892 were 0.003, 0.011, and 0.003 respectively. Since the frequencies of these three SNPs were so low in our cohort they were excluded from further analyses. The frequencies of the minor allelic variant for each of the remaining SNPs within the whole cohort were 0.111, 0.099, and 0.473 for rs4238001, rs61932577, and rs5888, respectively. All three SNPs were in Hardy−Weinberg equilibrium. Allele frequencies did not differ statistically by gender.

Table 3 shows the frequencies of the SR‐BI SNP genotypes and alleles grouped by HCV RNA status and Ishak fibrosis stage for chronically infected HCV subjects. LD analysis indicated that rs4238001 was not linked to either rs61932577 (D' = 0.075, *r* ^2^ = 0.005) or rs5888 (D' = 0.053, *r* ^2^ = 0.0004). Rs61932577 was found to be linked to rs5888 using the D' parameter of LD (D′ = 0.766). However, since the allele frequencies of rs61932577 and rs5888 differ widely the *r* ^2^ parameter has more desirable statistical properties,^52^ and this value does not indicate linkage between the two SNPs (*r* ^2^ = 0.059).

**Table 3:** SR‐BI SNP genotypes and allele frequencies

		HCV RNA		HCVIshak fibrosis	HCVIshak fibrosis
SRB1 genotype		Positive	Negative	Severe	Mild
		*n* = 267	*n* = 107	*n* = 92	*n* = 160
rs4238001	GG	211 (79.0)	85 (79.4)	70 (76.1)	130 (81.3)
	GA	52 (19.5)	20 (18.7)	22 (23.9)	27 (16.9)
	AA	4 (1.5)	2 (1.9)	0 (0.0)	3 (1.9)
rs61932577	CC	217 (81.3)	80 (74.8)	74 (80.4)	129 (80.6)
	CT	48 (18.0)	27 (25.2)	17 (18.5)	30 (18.8)
	TT	2 (0.7)	0 (0.0)	1 (1.1)	1 (0.6)
rs5888	CC	70 (26.2)	37 (34.6)	19 (20.7)	48 (30.0)^a^^,^ ^b^
	CT	137 (51.3)	53 (49.5)	45 (48.9)	84 (52.9)
	TT	60 (22.5)	17 (15.9)	28 (30.4)	28 (17.5)

				
SRB1 alleles		***N*** = **534**	***N*** = **214**	***N*** = **184**	***N*** = **320**
rs4238001	G	474 (88.8)	190 (88.8)	162 (88.0)	287 (89.7)
	A	60 (11.2)	24 (11.2)	22 (12.0)	33 (10.3)
rs61932577	C	482 (90.3)	187 (87.4)	165 (89.7)	288 (90.0)
	T	52 (9.7)	27 (12.6)	19 (10.3)	32 (10.0)
rs5888	C	277 (51.9)	127 (59.4)	83 (45.1)	180 (56.3)^c^
	T	257 (48.1)	87 (40.6)	101 (54.9)	140 (43.7)

Abbreviations: *n*, number of subjects; *N*, number of alleles; percentages are given in brackets.

^a^ Genotype versus disease severity *p* = 0.039 (*χ* ^2^ test);

^b^ Homozygous genotype versus disease severity *p* = 0.014 (*χ* ^2^ test);

^c^ Allele frequency versus disease severity *p* = 0.016 (*χ* ^2^ test).

For subjects with a history of HCV infection, there were no differences observed in the frequencies of rs4238001 and rs61932577 variants between subjects who had cleared the virus versus subjects with chronic infection or between subjects with severe versus mild liver disease. The rs5888 variant allele T was higher in HCV RNA positive subjects (48.1%) compared to those who had cleared the virus (40.6%) but this difference was not statistically significant (*χ* ^2^ test, *p* = 0.06). Genotype frequencies for the rs5888 SNP differed between chronically infected HCV subjects with severe and mild liver disease (*χ* ^2^ test, *p* = 0.039) and the variant allele T was found at a higher frequency in subjects with severe liver disease compared to those with mild liver disease (*χ* ^2^ test, *p* = 0.016). Since the frequency of rs5888 SNP heterozygotes was similar between the two groups of chronically infected subjects, analysis of homozygous genotypes only was performed. The frequency of homozygous genotype TT was higher in subjects with severe liver disease compared to subjects with mild liver disease (*χ* ^2^ test, *p* = 0.014).

Logistic regression analysis was performed to adjust for gender, age at biopsy, estimated duration of infection, and alcohol intake as confounding factors. Since the duration of infection was estimated from the date of exposure to risk, this excluded 56 patients with missing risk factor data from the analysis. Unadjusted and adjusted odds ratios are given in  Table 4. There was a significant association between the severity of liver disease and the rs5888 genotype TT, compared to genotype CC, after adjusting for the confounding factors with an adjusted odds ratio of 3.32 (95% confidence interval (CI) 1.05–10.53. *p* = 0.041).

**Table 4:** Logistic regression analysis of rs5888 genotype frequencies and the severity of liver disease

rs5888 Genotype	Unadjusted				Adjusted^a^			
	OR	95% CI		*p*	OR	95% CI		*p*
		Lower	Upper			Lower	Upper	
CC	1.00				1.00			
CT	1.35	0.71	2.57	0.356	1.52	0.60	3.93	0.384
TT	2.53	1.20	5.33	0.015	3.32	1.05	10.53	0.041

*Note*: exon 8 genotype frequencies are from 132 patients with mild and 46 subjects with severe liver disease.

^a^ Adjusted for gender, age at biopsy, estimated duration of infection and alcohol intake.

Haplotype analysis was performed to increase the statistical power of single locus analyses. All combinations of haplotypes of rs4238001, rs61932577 and rs5888 SNPs were analyzed. Haplotypes occurring at a frequency of 0.10 or more in both severe and mild HCV subjects are shown in Table 5. Haplotype rs4238001/rs61932577/rs588 G/C/C showed the most significant association with disease severity and was present at higher frequencies in subjects with mild liver disease compared to severe liver disease (*p* = 0.009). When the rs4238001 SNP was excluded from the haplotype, the frequency of rs61932577/rs5888 haplotype C/C was higher in patients with mild liver disease (*p* = 0.012) and haplotype C/T was found more frequently in subjects with severe liver disease (*p* = 0.015). rs4238001/rs5888 haplotype G/C was observed at a higher frequency in subjects with mild liver disease compared to severe liver disease (*p* = 0.018). Subjects who are heterozygous at multiple SNP loci cannot be unequivocally assigned a haplotype therefore subjects homozygous for SNP haplotypes were compared between the severe and mild liver disease groups using logistic regression. Since haplotypes containing the rs5888 SNP were consistently associated with disease severity only haplotypes containing rs5888 were compared. Unadjusted and adjusted odds ratios and CIs are shown in Table 6. After adjusting for gender, age at biopsy, estimated duration of infection and alcohol intake, subjects who were homozygous for both rs61932577 C and rs5888 T showed the most significant association with severity of liver disease compared to other haplotypes with an adjusted odds ratio of 2.81 (95% CI 1.06−7.46. *p* = 0.038).

**Table 5:** Haplotype frequencies subjects grouped by HCV liver disease severity

SNP Haplotype			Frequency	
rs4238001	rs61932577	rs5888	Severe	Mild
G	C	C	0.31	0.43**
	C	C	0.35	0.48**
	C	T	0.55	0.42*
G		C	0.40	0.50*
G	C	T	0.48	0.39*
G		T	0.49	0.40*
G	C		0.79	0.81

*Note*: haplotypes present at a frequency of at least 0.10 in both severe and mild liver disease subjects are shown. Haplotypes are listed in order of decreasing association.

** *p* < 0.01;

* *p* ≤ 0.05.

**Table 6:** Logistic regression analysis of frequency of homozygous haplotypes and the severity of liver disease

Haplotype^a^				Unadjusted				Adjusted^b^			
				OR	95% CI		*p*	OR	95% CI		*p*
rs4238001	rs61932577	rs5888	*n*		Lower	Upper			Lower	Upper	
G	C	C	38	0.58	0.27	1.25	0.16	0.64	0.21	2.02	0.45
	C	C	47	0.54	0.26	1.09	**0.09**	0.46	0.16	1.37	0.17
	C	T	54	2.26	1.22	4.16	**0.009**	2.81	1.06	7.46	**0.038**
G		C	51	0.60	0.30	1.18	0.14	0.74	0.29	1.86	0.52
G	C	T	40	2.20	1.10	4.35	**0.024**	2.37	0.78	7.20	0.13
G		T	42	1.96	1.00	3.82	**0.049**	1.91	0.65	5.60	0.24

Abbreviations: CI, confidence interval; n, number of subjects with homozygous haplotype; OD, odd ration.

^a^ Homozygous haplotype versus all other haplotypes present;

^b^ Adjusted for gender, age at biopsy, estimated duration of infection and alcohol.

Finally, since previous studies have reported gender‐specific effects of SR‐BI polymorphisms,^31^, ^32^ analysis of the cohort split by gender was undertaken. When the data obtained for the male patients was analyzed separately, a stronger association of the rs5888 polymorphism with disease severity was observed compared to the analysis for the full cohort (*n* = 174, *χ* ^2^ test, *p* = 0.011). The rs5888 allele T was found at a higher frequency in male subjects with severe liver disease (55%) compared to those with mild liver disease (40%) (*χ* ^2^ test, *p* = 0.006). By contrast, analysis of the association of the rs5888 allele on disease severity in females did not reveal any significant associations, with T allele frequencies of 54% and 53% in those with severe or mild disease, respectively (*n* = 75, *χ* ^2^ test, *p* = 0.927). Using logistic regression analysis to account for age at biopsy, estimated duration of infection and alcohol intake as confounding factors, carriage of rs5888 genotype TT was associated with an increased risk of severe disease compared to genotype CC in men, with an odds ratio of 4.38 (95% CI 1.05−18.27, *p* = 0.043). Similarly, haplotype analysis showed that the association of the haplotype G/C/C with disease severity was greater for the male data (*p* = 0.004) (Supporting Information: Table 2) than for the whole cohort.

### 3.3 SR‐BI polymorphisms and haplotypes are associated with differing SR‐BI mRNA expression

**Figure 1:**
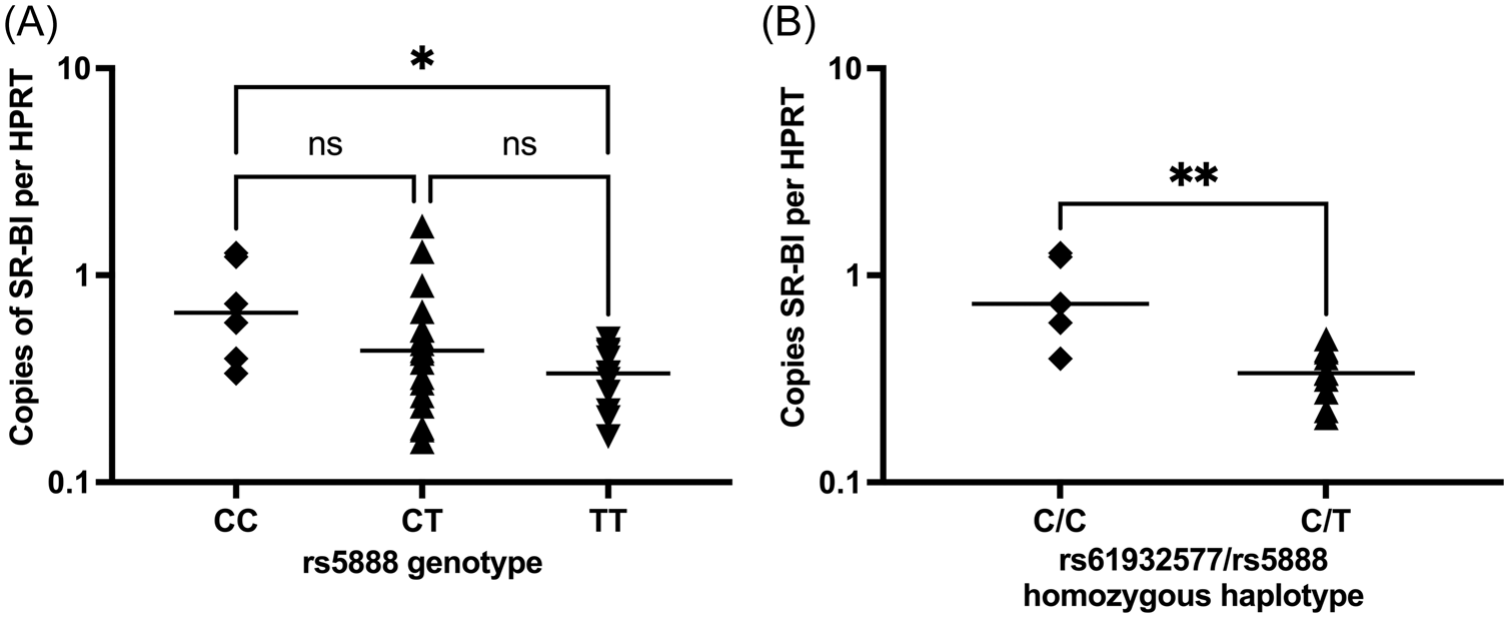
mRNA copies of SR‐BI per HPRT for SR‐BI variant rs5888 genotypes and haplotype. Log scale expression of SR‐BI per HPRT for rs5888 genotypes CC, CT and CC (A) and rs61932577/rs5888 homozygous haplotypes; C/T and C/C (B). Lines represent the median of the data set and data are representative of at least 2 repeats for each data point. *p < 0.05, **p < 0.01. Statistical comparisons performed were the Kruskal−Wallis ANOVA test with Dunn's multiple comparisons tests (A) or the Mann−Whitney test (B). ANOVA, analysis of variance.

The SR‐BI polymorphism associated with disease severity was a synonymous nucleotide substitution. To assess whether this polymorphism was exerting an effect through altered gene transcription SR‐BI RNA expression levels were determined in freshly obtained liver biopsies. *C*_t_ values were plotted against starting copy number to construct a standard curve from which to interpolate copy numbers of unknown samples. Using the standard curves of SR‐BI and HPRT the number of copies of SR‐BI and HPRT in the liver biopsy samples was determined and SR‐BI per HPRT RNA copy number plotted according to the SNP genotype and haplotype (Figure 1). Analysis of the median copies of SR‐BI per HPRT between rs5888 genotypes showed that rs5888 genotype CC was associated with twofold more SR‐BI expression compared to genotype TT (*n* = 17, Dunn's post‐test following Kruskal−Wallis test, *p* = 0.048). Since a SR‐BI homozygous haplotype consisting of rs61932577 C and rs5888 T was previously found to be associated with increased liver disease severity in chronically infected HCV subjects, analysis of SR‐BI mRNA levels grouped by the same homozygous haplotype was performed. Carriers of the homozygous intron 5/exon8 haplotype C/C had a twofold higher expression of copies of SR‐BI per HPRT compared with C/T (*n* = 15, Mann−Whitney *U* test, *p* = 0.008).

## 4 DISCUSSION

The clinical outcome of HCV infection is variable and remains poorly understood. Susceptibility to infection or disease progression may be influenced by the host genetic background, and polymorphisms within several genes have been associated with differing disease progression.^6^, ^10^, ^11^, ^53^, ^54^, ^55^ Genetic polymorphisms within the SR‐BI gene locus have been associated with differing plasma lipid profiles and risk of atherosclerosis and coronary artery disease,^23^, ^28^, ^30^, ^31^, ^32^, ^35^, ^38^, ^56^, ^57^ however the impact of SR‐BI allelic variation on outcome of HCV infection is still only partially understood. Since SR‐BI is a putative receptor for HCV entry,^17^ we performed the present study to define possible associations between SR‐BI polymorphisms with HCV disease. Genotyping was performed using a probe extension assay, which has previously been shown to produce comparable data to other genotyping methods.^58^, ^59^ Genotyping errors were further minimized by direct sequencing of a subset of PCR products used in the multiplex genotyping analysis. The minor allelic variants of rs5889, rs2293440 and rs5892 were rare in our population. Whilst this is at odds with reported global minor variant allele frequencies, this discrepancy can be explained by the ethnic composition of our cohort. For example, the global minor allele frequency (MAF) for rs5889 is 11%, whereas the frequency in our cohort was only 0.3%, although this latter value accords with the MAF frequency reported in for the European Caucasian 1000 genome project (phase 3) data set reported in the Ensembl database.^46^ Total allele frequencies for the SNPs in rs4238001, rs61932577, and rs5888 were similar to those reported elsewhere.^23^ Previous studies have reported linkage between the rs61932577 and rs5888 alleles.^23^, ^32^ A similar analysis of our data performed using the D′ parameter also indicated linkage of these two alleles. However, the *r* ^*2*^ value has more desirable properties.^60^, ^61^ A *r* ^*2*^ value of 1 has a stricter interpretation compared to D′, as *r* ^*2*^ can only equal 1 when the marker loci have identical allele frequencies and every occurrence of an allele at each of the markers perfectly predicts the allele at the other locus. D′ can equal 1 even when the allele frequencies vary widely.^52^ Reanalysis of our data using the more stringent *r* ^*2*^ value failed to show linkage between any of the alleles under study.

Our main finding was that the SR‐BI rs5888 polymorphism is associated with severity of liver fibrosis in chronic HCV infection. Since advanced liver fibrosis is a key factor for hepatocellular carcinoma and liver disease outcome,^62^ this finding is likely clinically relevant. We chose the Ishak score since Ishak fibrosis stage has been shown to predict clinical outcomes, need for liver transplantation, and liver‐related death in patients with chronic hepatitis C.^63^ Even in a background of confounding factors, such as age, alcohol consumption and duration of infection,^1^, ^2^, ^4^, ^5^ the rs5888 SNP allelic variant was associated with increased fibrosis. The effect of the rs5888 polymorphism was more pronounced when the data obtained for males was analyzed separately, but analysis of the female data set was not feasible due to low numbers. Analysis of a larger cohort of male and female HCV infected individuals would be necessary to elucidate the role of gender in defining the effects of this polymorphism in disease progression. Similarly, haplotype analyses using COCAPHASE for case‐control data^50^ showed that inheritance of a haplotype consisting of rs61932577 allele C and rs5888 allele T was significantly associated with increased risk of severe liver disease development.

To determine whether the rs5888 could be exerting an effect at the transcriptional level, SR‐BI mRNA was quantified in liver biopsy samples of chronically infected HCV subjects. The rs5888 minor variant homozygous genotype was associated with reduced SR‐BI mRNA levels. One previous study of the impact of SR‐BI variation on HCV^42^ reported reduced liver‐associated SR‐BI protein‐expression for the rs5888 TT genotype (together with an intronic variant, rs3782287, which we did not include in our study). Whilst these differences did not reach statistical significance, their sample size was relatively small (*n* = 40). The same paper highlighted that both the rs5888 CT heterozygote and minor homozygous TT genotypes were associated with significantly increased HCV viral load compared with the CC genotype. Unfortunately, viral load data for our historic cohort was not available, so we were unable to assess this relationship.

The association between rs5888 minor variant with decreased SR‐BI mRNA transcripts and increased liver disease severity was even more pronounced when considered in the context of the rs5888/rs61932577 haplotype. The study by Westhaus and colleagues^42^ also reported a similar increased association between reduced viral load when assessing rs5888 in the context of a haplotype containing a different intron 5 variant rs3782287. (This variant alone was significantly associated with increased viral load, and a trend toward lower liver SR‐BI protein expression.) Our combined data are comparable, and both show strong associations of SR‐BI variants and haplotypes with key outcomes of HCV infection as well as SR‐BI expression.

The mechanism by which reduced SR‐BI mRNA and protein expression might lead to increased HCV viral load and disease severity is unclear. Downregulation of SR‐BI expression on Huh‐7 cells by RNA silencing reduces HCVpp infectivity,^19^ therefore in vitro evidence would suggest decreased mRNA levels should result in lower HCV infectivity. However, such an effect would be less marked for HCV variants that are less dependent on SR‐BI for direct cell‐to‐cell spread.^64^ In addition, SR‐BI mRNA levels have been found to correlate with HCV RNA levels in the liver.^65^ However, viraemia can be present even in the absence of liver damage^66^; therefore the correlation of SR‐BI mRNA levels with HCV RNA levels may not necessarily be important in the severity of liver disease. Reduced SR‐BI transcription is associated with an increase in levels of HDL^22^, ^29^, ^67^ and the HDL has recently been shown to significantly enhance HCVpp entry through interaction with SR‐BI.^68^ Therefore, decreased SR‐BI transcription may lead to increased HDL levels, ultimately resulting in increased HCV infectivity. In this scenario, levels of receptor expression are not as important as the availability of HDL to potentiate the uptake of viral particles by hepatocytes.

An alternative hypothesis is that levels of plasma lipoproteins per se are more important in determining the severity of liver disease than the direct interaction of the virus with SR‐BI. Patients with chronic liver diseases often have impaired lipid metabolism.^69^ Cicognani et al.^69^ showed that patients with cirrhosis had reduced LDL, HDL and total cholesterol serum levels compared to subjects with coronary artery disease and normal controls. In these patients a decrease in LDL was related to increasing liver disease severity. The rs5888 T variant of SR‐BI has also been associated with reduced LDL‐C levels in large populations of healthy subjects.^31^, ^32^ The rs5888 polymorphism may therefore be a marker for altered levels of serum lipoproteins, augmenting an already existing consequence of severe liver disease, thereby resulting in more severe liver disease. Analysis of serum lipoprotein levels in the cohort studied here, together with cohorts of individuals with non‐HCV liver disease would be necessary to determine whether this polymorphism may be exerting an effect via alterations in lipoprotein levels. Furthermore, analysis of SR‐BI protein levels, relative to mRNA levels will be necessary to determine whether the rs5888 SNP variant influences receptor expression at the cell surface.

**Figure 2:**
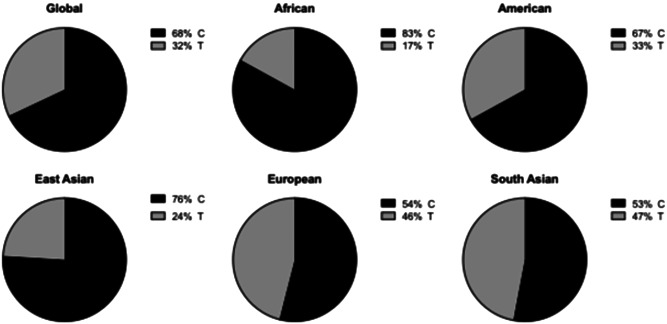
Frequency of rs5888 alleles in different populations. Data was obtained from the latest Ensembl release of SR‐B1 variant data derived from Phase 3 of the 1000 Genomes project.^46^ The relative frequency of each allele is represented in pie charts for individuals living in each geographical location.

The allele frequency for the rs5888 minor variant in our HCV cohort was 46%. Our cohort consists of predominantly white British people, and this MAF is similar to that reported in Phase 3 of the 1000 Genomes Project for British people living in Scotland and England (47%), and identical to that reported in European data set.^46^ However, it is important that additional studies investigating the potential association between this, and other SR‐BI variants are carried out in additional populations with different ethnic mixes; especially considering the major differences in MAF observed in the 1000 Genome Project data set (Figure 2).

In conclusion the rs5888 polymorphism variant is associated with an increased risk of severe liver disease in chronic HCV infection. Chronically infected HCV patients who are carriers of the rs5888 polymorphism were found to have reduced SR‐BI mRNA levels in liver biopsies. The mechanism whereby reduced SR‐BI mRNA and protein expression in the liver might lead to increased viral load and severity of liver disease will need to be elucidated. An assessment of any association with rs5888 (and its haplotypes) in other chronic liver diseases will provide at least some insight into the relative contribution of viral factors or lipid metabolism in disease pathogenesis. The data presented here support the involvement of SR‐BI in HCV infection and show that SR‐BI polymorphisms are important genetic predictors of liver disease progression in HCV infection.

## AUTHOR CONTRIBUTIONS

*Conceptualization*: Jonathan K. Ball. *Data curation*: Victoria L. Arandhara. *Formal Analysis*: Victoria L. Arandhara, Sally Chappell, Kevin Morgan, and Jonathan K. Ball. *Funding acquisition*: Jonathan K. Ball, William L. Irving, and Thomas F. Baumert. *Investigation*: Victoria L. Arandhara, Sally Chappell, and C. Patrick McClure. *Methodology*: Victoria L. Arandhara, C. Patrick McClure, and Alexander W. Tarr. *Resources*: William L. Irving. *Supervision*: Jonathan K. Ball and C. Patrick McClure. *Writing – original draft*: Victoria L. Arandhara and Jonathan K. Ball. *Writing – review and editing*: All authors.

## CONFLICT OF INTEREST

The authors declare no conflict of interest.

## Supporting information

Supplementary information.

Supplementary information.

## Data Availability

The data that support the findings of this study are available from the corresponding author upon reasonable request.
