# Supplementary material for: Scavenger receptor class B type I genetic variants associated with disease severity in chronic hepatitis C virus infection
Source: J Med Virol. 2022 Nov 30;95(1):e28331. doi: 10.1002/jmv.28331 (PMC10100136; doi:10.1002/jmv.28331)
Supplement: Supplementary file 1 — Supplementary information. [file JMV-95-0-s002.docx]

**Supplementary Table 1. PCR and SNaPshot Primer**
